# Genome-Wide Identification and Expression Analysis of the KUP Family under Abiotic Stress in Cassava (*Manihot esculenta* Crantz)

**DOI:** 10.3389/fphys.2018.00017

**Published:** 2018-01-24

**Authors:** Wenjun Ou, Xiang Mao, Chao Huang, Weiwei Tie, Yan Yan, Zehong Ding, Chunlai Wu, Zhiqiang Xia, Wenquan Wang, Shiyi Zhou, Kaimian Li, Wei Hu

**Affiliations:** ^1^Tropical Crops Genetic Resources Institute, Chinese Academy of Tropical Agricultural Sciences, Danzhou, China; ^2^Wuhan Centre for Disease Prevention and Control, Wuhan, China; ^3^College of Life Science and Technology, Huazhong University of Science and Technology (HUST), Wuhan, China; ^4^Key Laboratory of Biology and Genetic Resources of Tropical Crops, Institute of Tropical Bioscience and Biotechnology, Chinese Academy of Tropical Agricultural Sciences, Haikou, China; ^5^Hubei Key Laboratory of Purification and Application of Plant Anticancer Active Ingredients, Chemistry and Biology Science College, Hubei University of Education, Wuhan, China

**Keywords:** cassava, drought stress, gene expression, identification, KUP family

## Abstract

*KT/HAK/KUP* (KUP) family is responsible for potassium ion (K^+^) transport, which plays a vital role in the response of plants to abiotic stress by maintaining osmotic balance. However, our understanding of the functions of the KUP family in the drought-resistant crop cassava (*Manihot esculenta* Crantz) is limited. In the present study, 21 cassava *KUP* genes (*MeKUPs*) were identified and classified into four clusters based on phylogenetic relationships, conserved motifs, and gene structure analyses. Transcriptome analysis revealed the expression diversity of cassava *KUPs* in various tissues of three genotypes. Comparative transcriptome analysis showed that the activation of *MeKUP* genes by drought was more in roots than that in leaves of Arg7 and W14 genotypes, whereas less in roots than that in leaves of SC124 variety. These findings indicate that different cassava genotypes utilize various drought resistance mechanism mediated by *KUP* genes. Specific *KUP* genes showed broad upregulation after exposure to salt, osmotic, cold, H_2_O_2_, and abscisic acid (ABA) treatments. Taken together, this study provides insights into the *KUP*-mediated drought response of cassava at transcription levels and identifies candidate genes that may be utilized in improving crop tolerance to abiotic stress.

## Introduction

Potassium ion (K^+^) is an essential nutrient for various plant physiological functions, such as maintaining intracellular osmolality, cell turgor, and pH homeostasis. Although K^+^ is abundant on earth, the concentrations of K^+^ on the root surface is often lower than that in soil solution. Thus, plants depend on various K^+^ transport systems that mediate K^+^ uptake and transport to different plant tissues (Gierth et al., 2005; Ashley et al., 2006; Amrutha et al., 2007; Very et al., 2014). K^+^ transporters in plants can be classified into four major families: Trk/HKT, KT/HAK/KUP (KUP), CHX (cation/hydrogen exchanger), and KEA (K^+^ efflux antiporter) (Gupta et al., 2008). The KT/HAK/KUP family is the largest K^+^ transporter family and is responsible for K^+^ transport across membranes in bacteria, fungi, and plants (Li et al., 2017). The plant *KT/KUP/HAK* transporters were first isolated from barley (*HAK1*) and Arabidopsis (*KUP1/KT1* and *KUP2/KT2*) according to their homologs to fungal *HAK* and bacterial *KUP*. Thus, the composite name of *KT/HAK/KUP* is used to widely represent the entire family in plants (Very et al., 2014). Mutation analysis has revealed that the 8th transmembrane domain and the C-terminus of KT/HAK/KUP play crucial roles in determining K^+^ transport capacity (Mangano et al., 2008). Moreover, plant KT/HAK/KUPs have different K^+^ affinity and are involved in cation influx and efflux (Nieves-Cordones et al., 2016). These KT/HAK/KUPs contain 10–15 transmembrane domains with both N- and C-termini at the intracellular side of the membrane, the latter being much longer (Gierth and Maser, 2007; Nieves-Cordones et al., 2016). Molecular evolution analysis has indicated that segmental duplications occurred 35.89–62.77 million years ago, resulting in its expansion in tomato (Hyun et al., 2014). Phylogenetic analysis has grouped plant KT/HAK/KUPs into four clusters (Rubio et al., 2000; Banuelos et al., 2002). Most of the KUPs in cluster I function in high-affinity K^+^ uptake, whereas cluster II are involved in low-affinity K^+^ transport (Gupta et al., 2008). To date, 13 and 27 *KT/HAK/KUP* gene family members have been identified from Arabidopsis and rice, respectively (Rubio et al., 2000; Maser et al., 2001; Banuelos et al., 2002; Gupta et al., 2008). Expression analysis suggests that most members of the Arabidopsis *KT/HAK/KUP* family expressed in the roots, siliques, leaves, and flowers. *AtHAK5* expression is induced under conditions of K^+^ deprivation. Ten *AtKT/KUPs* expressed in root hairs and five of them expressed in root tip cells, thereby implying their role in K^+^ uptake (Ahn et al., 2004). In rice, transcripts of 26 *OsHAK* genes were detected in at least 1 of the 27 tested tissues, and five genes were observed to be expressed in all tissues in all three genotypes (Gupta et al., 2008). Moreover, the expression of *KT/HAK/KUP* genes in other plant species also supported their possible role in K^+^-mediated multiple biological processes, such as tissue development and abiotic stress responses (Su et al., 2002; Grabov, 2007; Song et al., 2015).

Some *KT/HAK/KUP* genes are essential for plant growth and development (Ahn et al., 2004). Knocking out *AtKT3/KUP4* results in tiny root hairs, suggesting its function in cell expansion (Rigas et al., 2001). A mutation in *AtKT/KUP2* (*shy3-1*) induces a dwarf phenotype, which results from a reduction in cell size (Elumalai et al., 2002). ARF2 directly bind to the promoter of HAK5, regulating root hair elongation (Zhao et al., 2016). Rice phloem has relatively high *OsHAK5* transcript levels that regulates K^+^/Na^+^ ratio during shoot growth (Yang et al., 2014). *OsHAK1* transcript abundance is elevated in the roots of K^+^-starved rice and *OsHAK1* mutants exhibit a reduction in root and shoot growth (Chen et al., 2015). The *GhKT1* was found to be associated with the expansion of cotton fibers in turgor-dependent growth (Ruan et al., 2001). *VvKUP1* and *VvKUP2* play a role in K^+^-mediated cell expansion in grape (Davies et al., 2006). These findings highlight the importance of KT/HAK/KUP transporters in plant development and K^+^ uptake.

Members of *KT/HAK/KUP* family also participate in stress-related responses. *OsHAK1* expression is induced by K^+^ deficiency or salt stress and it confers salt tolerance by regulating K^+^ uptake and K^+^/Na^+^ ratio (Chen et al., 2015). Knocking out *OsHAK21* decreases the K^+^/Na^+^ ratio and salt tolerance (Shen et al., 2015). Constitutive overexpression of *OsHAK5* in tobacco improves K^+^ accumulation during salt stress and confers increased salt resistance (Horie et al., 2011; Yang et al., 2014). *KUP2/6/8* plays a positive role in drought stress response by regulating osmotic homeostasis and the abscisic acid (ABA) response in Arabidopsis (Osakabe et al., 2013). Recently, INTEGRIN-LINKED KINASE1 (ILK1) was found to interact with and promote HAK5 accumulation and positively regulate osmotic stress tolerance in Arabidopsis (Brauer et al., 2016). Together, these studies reveal the crucial role of *KT/HAK/KUPs* in K^+^-mediated abiotic stress response.

Cassava (*Manihot esculenta* Crantz) is considered as a food crop and potential biofuel crop because of its high starch production (Zidenga et al., 2012). Cassava is highly resistant to abiotic stresses, such as drought and low nitrogen (Xu et al., 2013). Abiotic stress resistance in other crops may be improved using gene resources from cassava. However, the mechanism underlying cassava resistance to abiotic stress remains less known. Advancements in sequencing technologies have facilitated gene identification and expression analysis. We previously sequenced the genomes of different cassava subspecies (including wild ancestor species and modern cultivated species; Wang et al., 2014), which allows subsequent analysis of whole gene families in cassava.

To date, members of the *KT/HAK/KUP* family have been well-characterized in Arabidopsis, rice, peach, tomato, *Physcomitrella patens, Selaginella moellendorffii* and poplar by genome-wide analyses (Rubio et al., 2000; Maser et al., 2001; Banuelos et al., 2002; Gupta et al., 2008; Gomez-Porras et al., 2012; He et al., 2012; Song et al., 2015; Nieves-Cordones et al., 2016). In the present study, we identified 21 *KUP* genes (*MeKUPs*) from the cassava genome and analyzed their phylogenetic relationship, gene structure, conserved domain, and expression profiles in response to drought, salt, osmotic, cold, H_2_O_2_, and ABA treatments. Our analyses reveal the transcriptional control of *MeKUP* genes in different genotypes and candidate *KUP* genes that may be potentially utilized in improving crop resistance to abiotic stress.

## Materials and methods

### Plant materials and treatments

W14 (*M. esculenta* ssp. *flabellifolia*), a wild subspecies, is the nearest ancestor of cultivated cassava. It shows low photosynthesis rate, tuberous root yield, and starch content in tuberous root, but robust resistance to drought stress (Wang et al., 2014). KU50 is a representative cultivar of the cultivated cassava because of its high root yield and high starch content in tuberous root and extensively used in commercial plantations in East Asia (Utsumi et al., 2012; Wang et al., 2014). Arg7 is a variety containing elite agronomic traits, including a certain level of growth under moderate drought stress (Zhao et al., 2015). SC124, a widely planted cassava cultivar in China, can survive in prolonged severe drought stress (Zhao et al., 2015). Arg7, KU50, and W14 were used to study the expression profiles of *KUP* genes in different organs to get some clues on cassava organ development. W14 was confirmed to show stronger drought resistance than Arg7 and SC124 in our previous study (Hu et al., 2016). These three genotypes were selected to investigate the expression patterns of *KUP* genes in response to drought stress. Segments of cassava stems from mother plants were cultured in pots filled with soil and vermiculite (1:1) in growth room with a 16 h/35°C day and 8 h/20°C night regime, and a relative humidity of 70%. Thereafter, 90-day-old stems, 90-day-old leaves, 90-day-old tuberous roots (early), 150-day-old tuberous roots (middle), and 270-day-old tuberous roots (late) were acquired from KU50, Arg7, and W14 under normal conditions to study the expression levels of *MeKUPs* in distinct organs. To detect the transcriptional changes of *MeKUPs* in response to drought, leaves, and roots were collected from Arg7, SC124, and W14, respectively, under drought conditions for 12 d. For osmotic, salt, cold, ABA, and H_2_O_2_ treatments, 2-month-old seedlings of Arg7 were challenged with 200 mM mannitol for 2 h, 6 h, 3 d, 14 d, 18 d, and 24 d, 300 mM NaCl for 2 h, 6 h, 3 d, 14 d, 18 d, and 24 d, low temperature (4°C) for 2, 5, 15 h, 48 h following 7 and 14 d recovery, 100 μM abscisic acid (ABA) for 2, 6, 10, 24, 48, and 72 h, 10% H_2_O_2_ for 2, 6, 10, 24, 48, and 72 h, respectively.

### Identification and evolutionary analysis

The protein sequences of AtKUPs in Arabidopsis and OsHAKs in rice were downloaded from UniPort and RGAP, respectively (Kawahara et al., 2013; The UniProt Consortium, 2015). The whole protein and nucleotide sequences of cassava were downloaded from the cassava genome database (Prochnik et al., 2012). The known KUP protein sequences were used to build HMM profiles that were employed to query the cassava dataset using HMMER software (Eddy, 2011; Finn et al., 2011). The identified KUPs from cassava were also validated by BLAST with KUPs from rice and *Arabidopsis* as queries. With the PFAM and CDD databases, the identified cassava KUPs were subjected to conserved domains validation (Marchler-Bauer et al., 2015; Finn et al., 2016). The evolutionary trees were constructed with the KUPs proteins from Arabidopsis, rice, and cassava using MEGA 5.0 and Clustal X2.0 softwares (bootstrap values for 1,000 replicates) (Larkin et al., 2007; Tamura et al., 2011).

### Sequence analysis

ExPASy proteomics server was used to predict the molecular weight (MW) and isoelectric points (pI) of cassava KUP family proteins (Gasteiger et al., 2003). MEME program was employed to identify the conserved protein motifs of MeKUPs, which were further annotated with InterProScan (Mulder and Apweiler, 2007; Brown et al., 2013). The gene structures were assessed with the GSDS software (Hu B. et al., 2015; Hu W. et al., 2015). The *cis*-elements in the 1500 bp sequences upstream of the coding sequences were analyzed by PlantCARE databases. Those elements (ABRE, DRE, LTRE, ERE, MBS, and GARE) related to abiotic stress response were subjected to further analysis (Shinwari et al., 1998; Narusaka et al., 2003; Gou et al., 2010; Yun et al., 2010).

### Transcriptome analyses

Total RNA of each sample was extracted with plant RNA extraction kit (TIANGEN, China) and used for cDNA library construction. The sequencing was performed with an Illumina GAII following manufacturer's instructions. Adapter sequences were removed with FASTX-toolkit. Clean reads were generated by removing low quality sequences using FastQC. Tophat v.2.0.10 was used to map the clean reads to the cassava genome. Using cufflinks, the transcriptome data was assembled (Trapnell et al., 2012). Reads Per Kilobase of exon model per Million mapped reads (FPKM) was employed to calculate gene expression levels. The transcriptiomic data was submitted to NCBI and the accession number was listed in Table S1.

### Quantitative real-time PCR (qRT-PCR) analysis

qRT-PCR analysis was run on StratageneMx3000P (Stratagene, CA, USA) instrument using SYBR®Premix Ex Taq™ (TaKaRa). The relative expression of the tested *MeKUP* genes under different treatments was measured according to 2^−ΔΔCt^ method (Livak and Schmittgen, 2001). The primer pairs were examined by melting curve, agarose gel electrophoresis, and sequencing PCR products (Table S2). The amplification efficiency was in the range of 0.92–1.04. The relative expression of *MeKUP* genes in each time point was calculated according to the control and treated samples that consist of three independent experiments.

## Results

### Identification of the KUP gene family in *Manihot esculenta*

**Figure 1:**
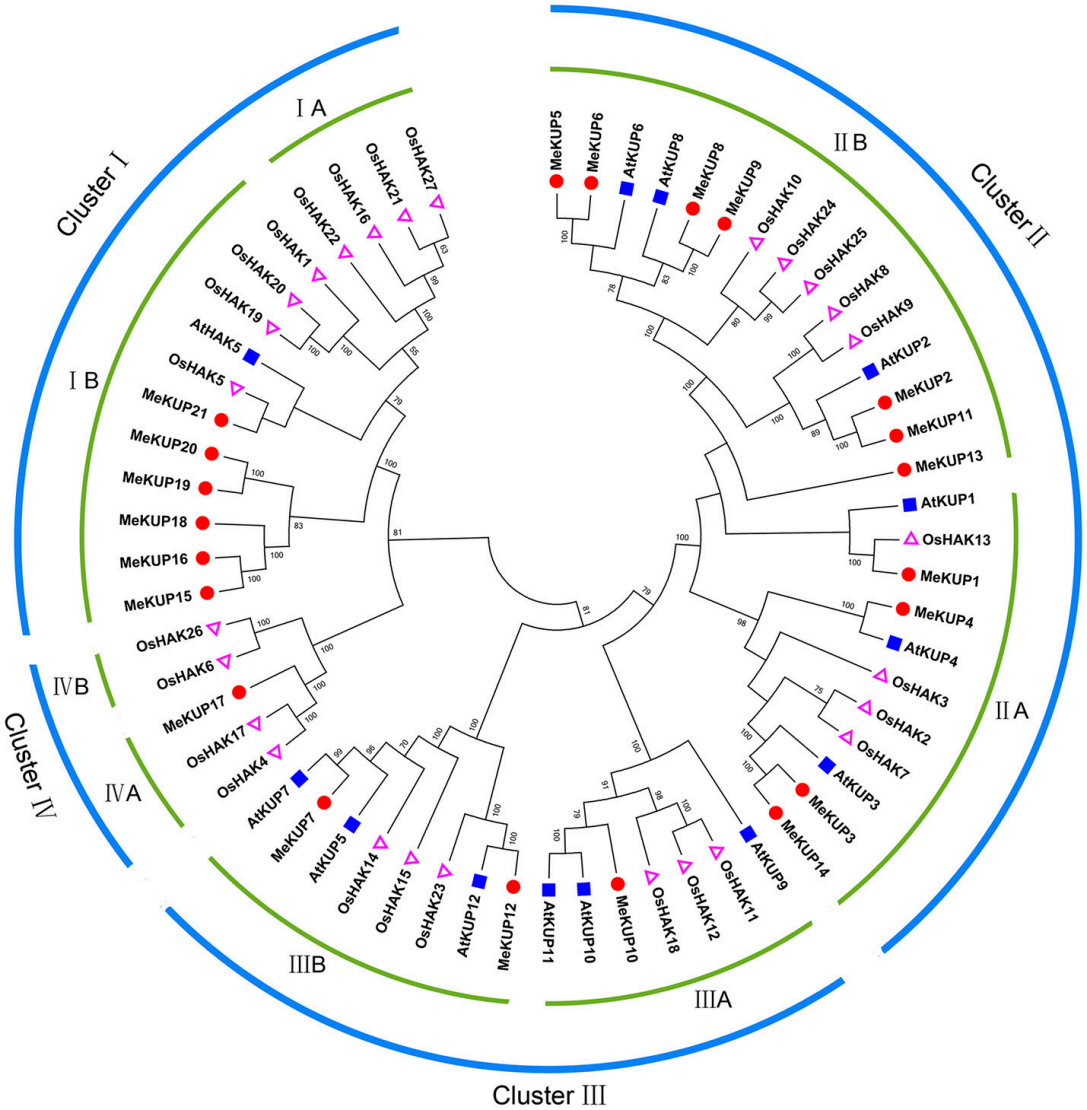
Phylogenetic analysis of KUPs from cassava, rice, and Arabidopsis using the complete protein sequences. The Neighbor-joining (NJ) tree was reconstructed using Clustal X 2.0 and MEGA 5.0 softwares with the pair-wise deletion option. One thousand bootstrap replicates were used to assess tree reliability.

BLAST and Hidden Markov Model searches were conducted to extensively identify cassava KUPs using Arabidopsis and rice KUP protein sequences as queries. Twenty-one predicted full-length MeKUPs were identified in the *M. esculenta* genome, which were designated as MeKUP1-MeKUP21 based on their phylogenetic relationship with Arabidopsis. Conserved domain analysis further confirmed that all KUPs contain one K^+^ potassium transporter domain, which is hallmark of the KUP family (Table S3). The number of amino acid residues of the predicted MeKUPs ranged from 572 to 840, and their relative molecular mass varied from 63.97 to 87.93 kDa (Table S4). Phylogenetic analysis of the 21 MeKUPs together with 13 AtKUPs and 27 OsHAKs showed that the KUP family could be classified into four clusters (from I to IV). Cluster I included MeKUP15,-16,-18,-19,-20, and -21; Cluster II consisted of MeKUP1,-2,-3,-4,-5,-6,-8,-9,-11,-13, and -14; Cluster III comprised MeKUP7,-10, and -12; and Cluster IV included MeKUP17 (Figure 1; Table S5). The constructed dendrogram showed that MeKUPs were generally most closely related to the KUPs of Arabidopsis than those of rice, which coincides with current established plant evolutionary relationships.

### Conserved motif analysis

**Figure 2:**
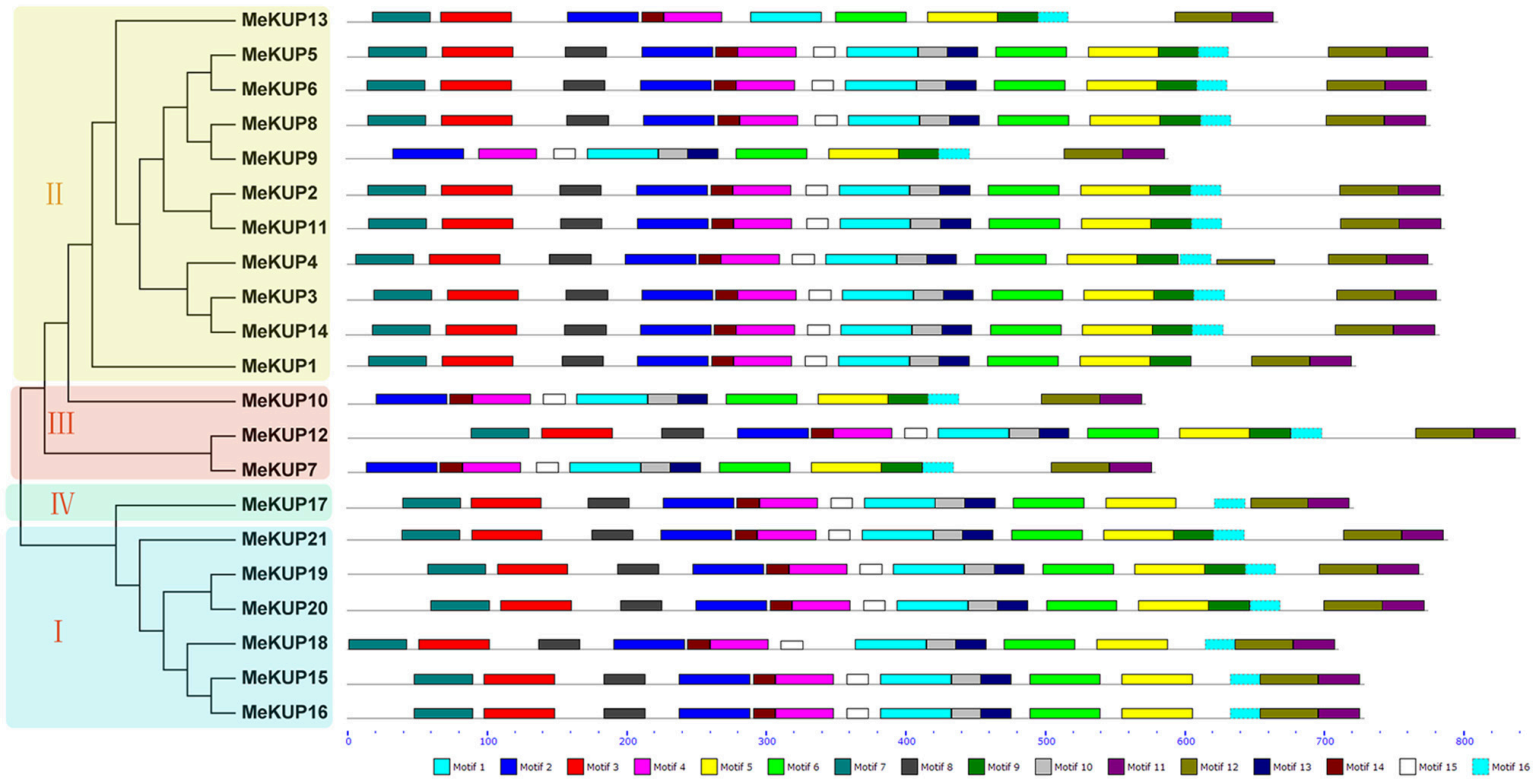
The conserved motifs of cassava KUPs according to phylogenetic relationship. All motifs were identified by MEME database with the complete amino acid sequences of cassava KUPs.

To study the structural features of the MeKUPs, conserved motifs were identified based on their evolutionary relationships. MEME database search identified 16 conserved motifs (Figure 2). After InterProScan search, motifs 1, 2, 3, 4, 6, 7, and 8 were annotated as K^+^ potassium transporter motif (Table S6). As shown in Figure 2, all the identified MeKUPs contained motifs 1, 2, 4, 5, 6, 11, and 12, suggesting that at least four K^+^ potassium transporter motifs existed in all 21 MeKUPs. The KUPs in Cluster I and IV harbored motifs 1–8 and 10–16. Cluster II KUPs showed motifs 1–16, except for MeKUP1, MeKUP9, and MeKUP13. Cluster III KUPs featured motifs 1–2, 4–6, and 9–16. Although some homologous KUPs had distinct motifs structures, such as MeKUP8/9 and MeKUP7/12, most of the homologous KUPs showed the same motif structure, including MeKUP5/6, MeKUP2/11, MeKUP3/14, MeKUP15/16, and MeKUP19/20. Together, these results indicate that all the identified MeKUPs have typical motifs of K^+^ potassium transporter, and each subgroup shares similar motif features, further supporting the phylogenetic classification of KUP family.

### Gene structure and promoter analysis

**Figure 3:**
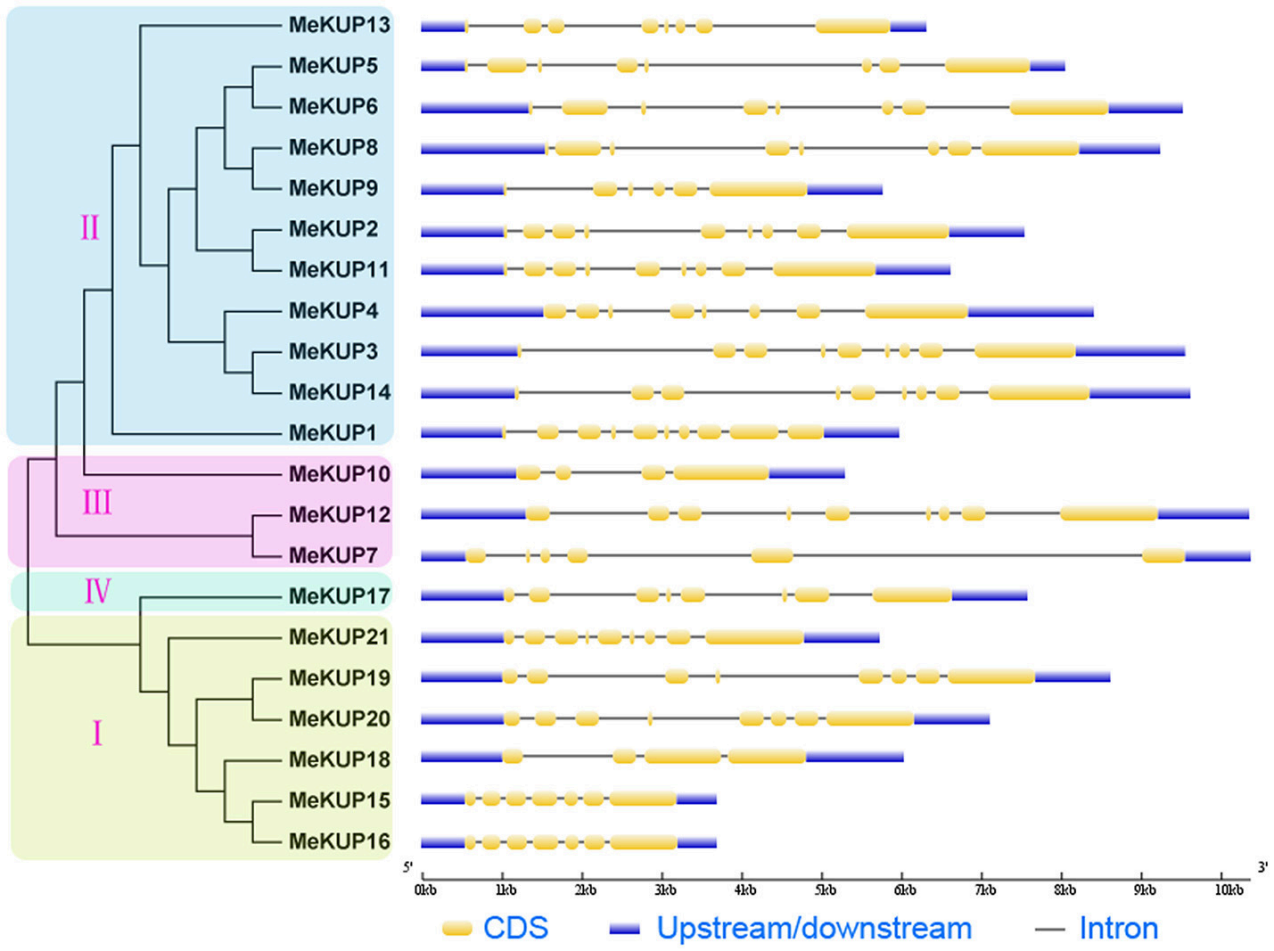
Gene structure analyses of cassava *KUPs* according to phylogenetic relationship. Exon-intron structure analyses were performed by GSDS database. The blue boxes, yellow boxes, and the black lines indicate upstream/downstream, exons, and introns, respectively.

As shown in Figure 3, although two *MeKUP* genes (*MeKUP10* and *MeKUP18*) had only four exons, the majority of the *MeKUP* genes harbored 6 to 10 exons. Additionally, some *MeKUP* genes in the same cluster had the same amount of exons such as *MeKUP19,-20*, and *-21* in Cluster I, and *MeKUP4,-5,-6,-8*, and *-13* in Cluster II. Furthermore, 1,500 bp upstream sequences of coding sequence from *MeKUPs* were identified, and the stress responsive *cis*-elements, including ABA-responsive element (ABRE), dehydration-responsive element (DRE), low temperature-responsive element (LTRE), ethylene-responsive element (ERE), MYB-binding site (MBS), and gibberellin-responsive element (GARE) in the *MeKUP* gene promoters were analyzed. The results revealed that 38.1% of *MeKUPs* contained ABRE, 23.8% contained LTRE, 19.0% contained ERE, 71.4% contained MBS, 28.6% contained GARE, and DRE was not found in all *MeKUPs*. From the above results, 85% of the MeKUPs (except for *MeKUP7, MeKUP18*, and *MeKUP20*) contained at least one of the tested elements in their promoter regions, suggesting the possible involvement of these genes in responses to different abiotic stressors.

### Expression profiles of *MeKUP* genes in different tissues

**Figure 4:**
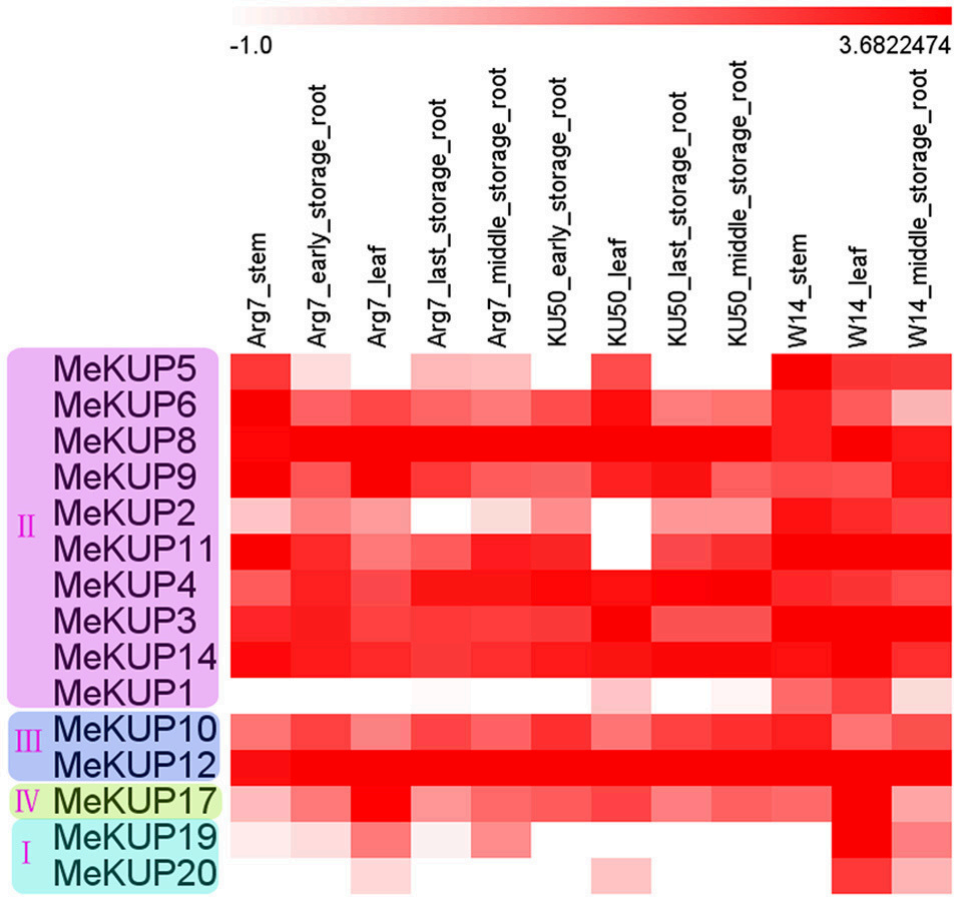
Expression profiles of cassava *KUPs* in different tissues of Arg7, KU50, and W14. Log2 based fold change was used to create the heat map. Fold changes in gene expression are shown in color as the scale.

To study the expression profiles of *MeKUP* genes in different tissues, transcriptome analyses of the leaves, stems, and storage roots in a wild subspecies (W14) and two cultivated varieties (Arg7 and KU50) were performed (Figure 4; Table S7). Fifteen *MeKUP* genes expressed in the tested tissues of the three genotypes. Moreover, *MeKUP3,-4,-8,-12*, and *-14* showed high expression levels (Log_2_ based value >2) in all organs of the three cassava varieties. However, *MeKUP1,-19*, and *-20* were highly expressed only in the leaves of W14. In addition, *MeKUP5* had broadly high expression in W14, and only highly expressed in the stems of Arg7 and the leaves of KU50. These results implied the differential roles of these genes in tissue development in different genotypes.

### Expression profiles of *MeKUP* genes in response to drought stress

**Figure 5:**
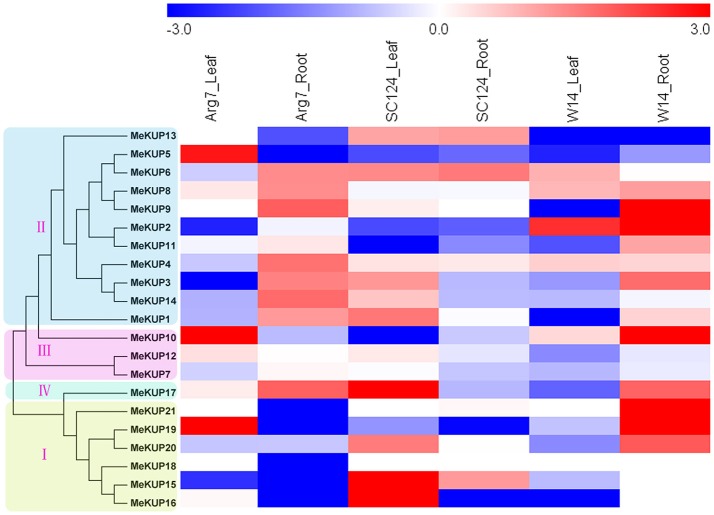
Expression profiles of cassava *KUPs* in response to drought stress in leaves and roots of Arg7, SC124, and W14. Log2 based fold change was used to create the heat map. Fold changes in gene expression are shown in color as the scale.

Because previous reports have revealed that the member of *KT/HAK/KUP* family participate in osmotic adjustment and drought stress response, the expression profiles of *MeKUP* genes in response to drought stress were further detected in three cassava genotypes by transcriptome analysis (Figure 5; Table S8). In the Arg7 variety, 3 and 8 of the 21 *MeKUP* genes were induced (Log_2_ based fold change >1) in leaves and roots after drought treatment. In the SC124 variety, 8 and 3 of the 21 *MeKUP* genes were upregulated (Log_2_ based fold change >1) in leaves and roots after drought treatment. In the W14 accession, 1 and 10 of the 21 *MeKUP* genes showed induction (Log_2_ based fold change >1) in leaves and roots after drought treatment. These results indicated that a higher number of *MeKUP* genes were upregulated in the roots in response to drought than that in leaves of Arg7 and W14, whereas fewer genes in roots than that in leaves of SC124 were induced after drought exposure. Generally, *MeKUP* genes show similar expression profiles in Arg7 and W14, which differs from that in SC124 after drought treatment. *MeKUP3* showed repression (Log_2_ based fold change < 1) in the leaves of Arg7 and W14, whereas induction (Log_2_ based fold change >1) in the leaves of SC124. *MeKUP-3,-8,-9*, and *-17* were upregulated (Log_2_ based fold change >1) in the roots of W14 and Arg7, whereas downregulated or no response in the roots of SC124.

Notably, 3 (*MeKUP-5,-10*, and *-19*), 3 (*MeKUP-15,-16*, and *-17*), and 1 (*MeKUP2*) KUP genes showed strong induction (Log_2_ based fold change >2) after drought stress in the leaves of Arg7, SC124, and W14 respectively. In contrast, 5 *KUP* genes (*MeKUP-2,-9,-10,-19*, and *-21*) were strongly upregulated (Log_2_ based fold change >2) in the roots of W14 after drought treatment, whereas no *KUP* genes were strongly induced after drought treatment in the roots of Arg7 and SC124. Thus, the total number of strongly upregulated *MeKUP* genes was higher in W14 than in Arg7 and SC124.

### Differential expression of *MeKUP* genes under abiotic stress and signal molecule treatments

**Figure 6:**
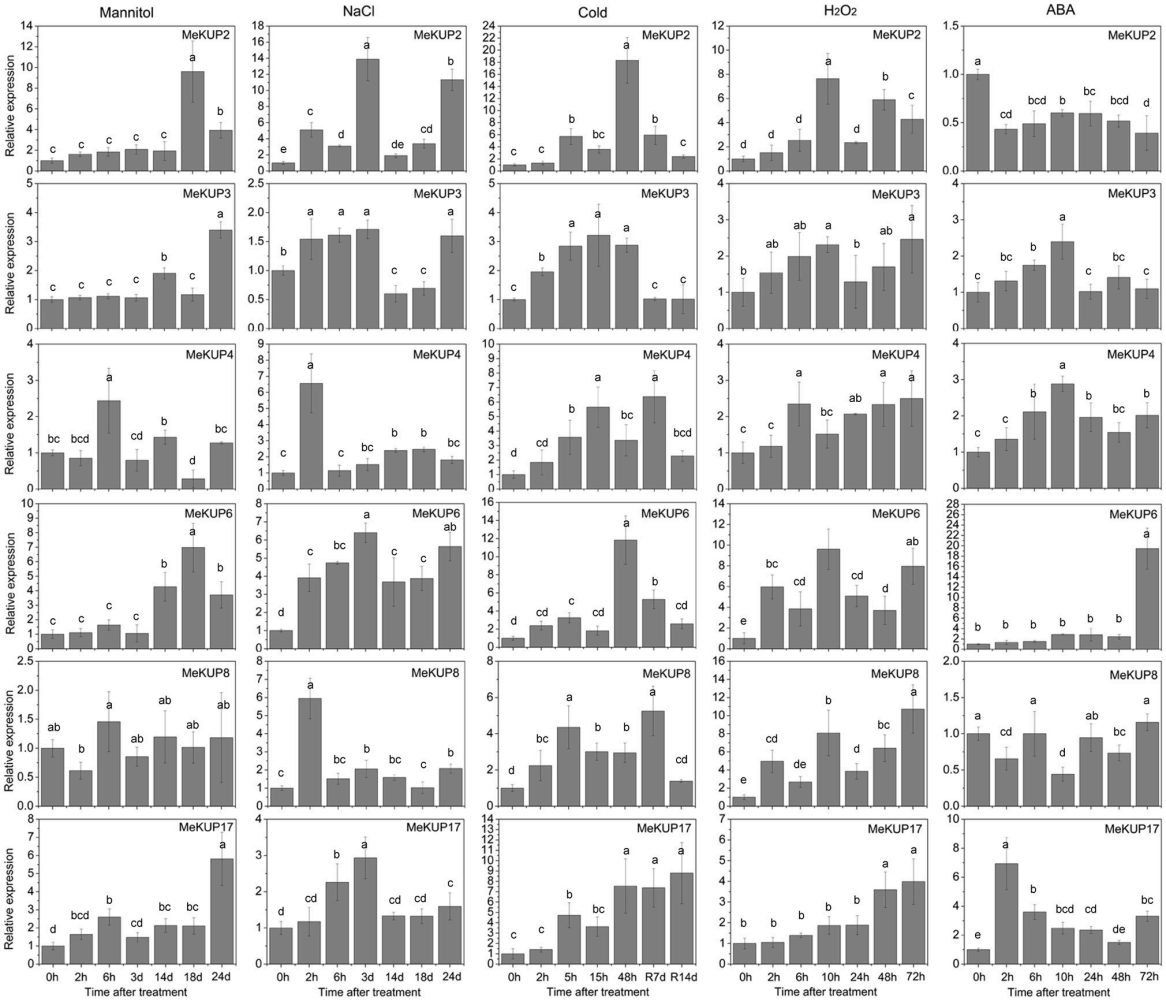
Expression patterns of *MeKUPs* after osmotic, NaCl, cold, H_2_O_2_, and ABA treatments treatment in cassava. The mean fold changes of each gene between treated and control samples at each time point were used to calculate its relative expression levels. NTC indicates no treatment controls (mean value = 1). Data are means ± *SD* of *n* = 3 biological replicates. Means denoted by the same letter do not significantly differ at *P* < 0.05 as determined by Duncan's multiple range test.

Based on the RNA-seq data, some *MeKUP* genes were upregulated in different cassava genotypes such as *MeKUP2* in the leaves and roots of W14, *MeKUP3* and *MeKUP17* in the roots of Arg7 and W14 and the leaves of SC124, *MeKUP4* in the roots of Arg7 and W14, *MeKUP6* in the roots of Arg7 and SC124 and leaves of SC124, and *MeKUP8* in the roots of Arg7 and W14. To investigate the response of *MeKUP* genes to abiotic stresses and related signaling at the transcriptional level, these six genes (*MeKUP-2,-3,-4,-6,-8*, and *-17*) were selected for further expression profiling after salt, osmotic, cold, H_2_O_2_, and ABA treatments (Figure 6). *MeKUP2* was upregulated after 18–24 d of exposure to osmotic stress, 2–24 d of exposure to salt stress, 5–48 h of cold treatment followed by 7 d of recovery, and 10–72 h of H_2_O_2_ treatment, whereas it was downregulated by ABA treatment. *MeKUP3* was upregulated after 2–3 d and 24 d of salt treatment, 14 and 24 d of osmotic treatment, 2–48 h of cold treatment, 2–10 h and 48–72 h of H_2_O_2_ treatment, and 2–10 h of ABA treatment. *MeKUP4* was induced after 6 h and 14 d of osmotic treatment, and upregulated by salt, cold, H_2_O_2_, and ABA treatments at most of the tested time points. *MeKUP8* was upregulated after $2 h-14 d and 24 d of salt treatment, 2–48 h of cold treatment and after recovery, and 2–72 h of H_2_O_2_ treatment, whereas downregulated after 2, 10, and 48 h of ABA treatment. In addition, *MeKUP6* and *MeKUP17* showed induction after all the treatments at several time points.

## Discussion

*KUP* genes play important roles in plant growth, development, and response to abiotic stresses (Osakabe et al., 2013; Zhao et al., 2016). Currently, our understanding of the role of KUP family in the drought-resistant crop cassava is limited. Here, we identified 21 KUPs from the cassava genome, which was classified into four clusters based on their evolutionary relationships (Figure 1). This is consistent with the previous classification of KUPs in Arabidopsis and rice (Rubio et al., 2000; Maser et al., 2001; Banuelos et al., 2002; Gupta et al., 2008). Moreover, the classification of cassava KUPs was further supported by conserved motif and gene structure analyses with each subgroup sharing similar motifs and exon-intron structures (Figures 2, 3). Conserved motif analysis suggested that all the identified KUPs had at least four typical motifs of K^+^ potassium transporters (Figure 2). In rice, all the 27 KUP members, except for OsHAK22, showed three conserved motifs (Gupta et al., 2008). Besides, some KUP proteins harbored a truncated K^+^ potassium transporter motif (He et al., 2012). This evidence indicates protein structure divergence among KUP family members over the course of evolution. Gene structure analysis showed that most of the *MeKUP* genes contained 6-10 exons, and the last exon exhibited the maximum length, which coincides with the exon-intron structure of KUP genes from Arabidopsis and poplar (Ahn et al., 2004; He et al., 2012). This indicates the conservation of the gene structure of the *KUP* family.

Organ expression profile analyses revealed 15 differentially expressed *MeKUP* genes in the stems, leaves, and storage roots of three cassava genotypes, including five (*MeKUP3,-4,-8,-12*, and *-14*) that showed high expression levels in all organs of the three cassava genotypes (Figure 4). In Arabidopsis, differentially expressed *KUP* genes were detected in the roots, older leaves, younger leaves, developing siliques, and flowers (Ahn et al., 2004). In rice, five *KUP* genes (*OsHAK2, OsHAK10, OsHAK15, OsHAK23*, and *OsHAK25*) expressed in all tissues of three genotypes (Gupta et al., 2008). In peach, *KT/HAK/KUP* genes expressed in nine tested tissues (Song et al., 2015). Additionally, three *MeKUP* genes (*MeKUP1,-19*, and *-20*) showed high expression only in the leaves of W14 (Figure 4). This phenomenon has also been observed in Arabidopsis and rice, with *OsHAK5, OsHAK16, OsHAK25, AtKUP6, AKUP8*, and *AtKUP9* showing high expression in the leaves. Besides, the fruits and leaves of peach showed the highest number of expressed *KT/HAK/KUP* genes (Song et al., 2015). K^+^ is an essential nutrient for various physiological processes, particularly plant growth, development, and responses to abiotic stress (Ruan et al., 2001; Ahn et al., 2004; Osakabe et al., 2013). The high expression of *KUP* genes in leaves suggests its involvement in K^+^ transport during leaf development or environment adaptation.

Previous studies have revealed the fundamental role of osmotic adjustment in plant response to drought stress. Cellular osmotic balance is affected by various substances, including amino acids, sugars, and K^+^ (Osakabe et al., 2013). K^+^ uptake and efflux involve various types of channels and transporters that regulate water potential and turgor during osmotic adjustment (Very and Sentenac, 2003). Biochemical and genetic studies further support that the *KUP/HAK/KT* family transporters, including *AtKUP2/6/8* and *OsHAK1/5/21*, positively regulate drought and osmotic resistances by influencing the K^+^-mediated ABA response, stomatal behavior, and osmotic homeostasis (Gierth and Maser, 2007; Grabov, 2007; Horie et al., 2011; Osakabe et al., 2013; Yang et al., 2014; Chen et al., 2015; Shen et al., 2015; Brauer et al., 2016). In the present study, we observed that drought stress induced the upregulation of several cassava *KUP* genes in the roots and leaves of different genotypes, suggesting their possible roles drought stress response. Generally, *MeKUP* genes showed similar expression profiles in Arg7 and W14, which differed from that in SC124 after drought treatment. The number of *MeKUP* genes upregulated by drought was significantly higher in the roots than that in the leaves of Arg7 and W14, whereas fewer genes were upregulated in the roots than in the leaves of SC124 (Figure 5). Previous studies have demonstrated that *KUP* genes positively regulate ABA response during lateral root formation and K^+^ efflux-mediated stomatal closure in leaves, thereby increasing plant resistance to drought and osmotic stresses (Osakabe et al., 2013). Based on this evidence, the expression diversity of *KUP* genes under drought stress implies differences in its roles in drought response in various cassava genotypes. For the Arg7 and W14 genotypes, a higher number of *MeKUP* genes are involved in drought-induced ABA responses in roots, whereas in SC124, more *MeKUP* genes participate in K^+^ efflux-mediated stomatal closure in the leaves under drought stress. Both functions contribute to cassava resistance to drought stress.

Additionally, the total number of strongly upregulated *MeKUP* genes (Log_2_ based fold change >2) was higher in W14 than in Arg7 and SC124 (Figure 5). W14 has greater drought resistance than Arg7 and SC124 (Hu et al., 2016). Previous studies have demonstrated that *KUP* genes play a positive role in drought or osmotic stress response by affecting ABA response, stomatal behavior, and osmotic homeostasis (Gierth and Maser, 2007; Grabov, 2007; Osakabe et al., 2013). These drought or osmotic responses involve *KUP*-mediated K^+^ uptake from the roots, transport from vascular tissues, and efflux from the leaves (Li et al., 2017). Thus, a higher number of strongly upregulated *MeKUP* genes in W14 may contribute to its drought resistance.

Previous studies have demonstrated the positive role of *KUP* genes in osmotic, drought, salt, or ABA responses, such as, *AtKUP2, AtKUP6*, and *AtKUP8* in Arabidopsis, and *OsHAK1, OsHAK5*, and *OsHAK21* in rice (Horie et al., 2011; Osakabe et al., 2013; Chen et al., 2015). Transcriptome analysis has identified several *MeKUP* genes that were responsive to drought stress (Figure 5). Thus, there is a need to investigate the expression patterns of *MeKUP* genes under various abiotic stress and stress-related signal molecule treatments. In the present study, we observed that all the tested genes (*MeKUP-2,-3,-4,-6,-8*, and *-17*) showed induction after salt and osmotic treatments at several time points, which coincides with the expression patterns of *AtKUP2/6/8* in Arabidopsis (Osakabe et al., 2013). Notably, *MeKUP2/6/8* is the homologs of *AtKUP2/6/8* according to the evolutionary analysis, thereby suggesting that it may also be involved in abiotic stress response. All tested cassava genes were induced after cold and H_2_O_2_ treatments, which supplies a clue for further investigation of KUP-mediated cold and H_2_O_2_ responses. *MeKUP6* and *MeKUP17* were upregulated after all the treatments, indicating that these genes may play a role in multiple stress signaling pathways (Figure 6). Based on the importance of K^+^-mediated osmotic adjustment in plant response to abiotic stress, the observed response of *MeKUP* genes under abiotic stress, and the nature of cassava resistance to drought, it may be important to functionally characterize these genes.

In conclusion, this study identified 21 KUPs in cassava and investigated their classification, protein motifs, and gene structure. Transcriptome analysis revealed the potential role of *MeKUP* genes against drought stress in different cassava genotypes. Additionally, we identified several *MeKUP* genes that may be utilized as candidates for improving crop resistances to multiple stresses. Further studies are required to reveal the molecular mechanisms of MeKUPs in response to abiotic stress at translational and post-translational levels by biochemical and genetic approaches.

## Author contributions

SZ, KL, and WH: conceived the study; WO, XM, CH, WT, YY, ZD, CW, ZX, and WW: performed the experiments and carried out the analysis; WH, WO, XM, and CH: designed the experiments and wrote the manuscript.

### Conflict of interest statement

The authors declare that the research was conducted in the absence of any commercial or financial relationships that could be construed as a potential conflict of interest.
